# Posterior spinal fusion to sacrum in non-ambulatory hypotonic neuromuscular patients: sacral rod/bone graft onlay method

**DOI:** 10.1007/s11832-014-0581-4

**Published:** 2014-04-13

**Authors:** Theresa Bui, Frederic Shapiro

**Affiliations:** Department of Orthopaedic Surgery, Boston Children’s Hospital, 300 Longwood Avenue, Boston, MA 02115 USA

**Keywords:** Spinal fusion, Sacral rod/bone graft onlay method, Scoliosis, Neuromuscular, Nonambulatory, Hypotonic

## Abstract

**Purpose:**

A retrospective study involving 65 non-ambulatory patients with hypotonic neuromuscular scoliosis has assessed the effectiveness of a sacral rod/bone onlay technique for extending spinal fusion to the sacrum.

**Methods:**

To extend posterior spinal fusion to the sacrum, we used either 1 Harrington rod and 1 Luque L rod with sublaminar wires in 14 patients (Group 1) or two rods with sublaminar wires in 51 patients (Group 2) along with abundant autograft and allograft bone covering the ends of the rods.

**Results:**

Diagnoses were Duchenne muscular dystrophy 53, spinal muscular atrophy 4, myopathy 3, limb girdle muscular dystrophy 2, infantile FSH muscular dystrophy 1, cerebral palsy 1, and Friedreich ataxia 1. Mean age at surgery was 14.3 years (±2.2, range 10.9–25.2). Radiographic follow-up (2 years post-surgery or greater) was 6.4 years (±4.4, range 2–25.3). Using the onlay technique, all patients fused with no rod breakage or pseudarthrosis. For the entire series, the mean pre-operative scoliosis was 54.7° (±31.1, range 0°–120°) with post-operative correction to 21.8° (±21.7, range 0°–91°) and long-term follow-up 24° (±22.9, range 0°–94°). For pelvic obliquity, pre-operative deformity was 17.3° (±11.3, range 0°–51°) with post-operative correction to 8.9° (±7.8, range 0°–35°) and long-term follow-up 10.1° (±8.1, range 0°–27°). Five required revision at a mean of 3.3 years post-original surgery involving rod shortening at the distal end. One of these had associated infection.

**Conclusion:**

Lumbosacral stability and long-term sitting comfort have been achieved in all patients. Problems can be minimized by positioning the rods firmly against the sacrum at the time of surgery with a relatively short extension beyond the L_5_–S_1_ junction. The procedure is valuable in hypotonic non-ambulatory neuromuscular patients whose immobility enhances the success rate for fusion due to diminished stress at the lumbosacral junction. It is particularly warranted for those with osteoporosis and a small, deformed pelvis. Considerable weight loss and lengthy rods not closely apposed to the sacrum at the time of surgery played a major role in patients needing revision.

## Introduction

The advantages of extending spinal fusion to the sacrum and pelvis in scoliosis surgery are considerable, providing greater stability and further limiting progression of deformity. This approach has been attempted since the beginning of spinal deformity surgery, even prior to the use of metallic stabilization [1–4]. With the use of internal fixation, many methods for extending fusion to the sacrum have been attempted, mainly by placing the metal rods within the sacrum and/or ilium or attaching them to bone by screw or hook fixation [1, 5–18]. As rigidity increased from extensive thoracolumbar stabilization, increasing stress was placed on the lumbosacral junction and on pelvic fixation devices, especially if limited to the sacrum. Results extending fusion to the pelvis improved, but the complication rate with infection, pain, less than full fusion, instrument failure, and metallic pullout was high, often approaching 40–50 % rates of pseudarthrosis [1, 5, 17–22]. In scoliosis surgery, in situ fusion and prolonged immobilization to achieve lumbosacral fusion is of historical interest, but currently has no place in attaining fusion at the lumbosacral junction which requires instrumentation to increase the fusion rate [1]. Several studies have assessed the complex anatomy of the pelvis [1, 5, 16] and the biomechanical aspects of fusion to the sacrum [1, 5, 8, 16, 23, 24]. In some neuromuscular patients, particularly with Duchenne muscular dystrophy (DMD), many surgeons have extended the fusion only to L_5_ in those with relatively mild deformity, bypassing pelvic concerns [25–36].

We report our experience with fusion to the sacrum in a specific group of adolescent non-ambulatory patients with hypotonic neuromuscular scoliosis. Owing to our concern about metallic fixation in the relatively thin, markedly osteopenic, and often deformed sacral and iliac bone, we specifically place the distal end of one or two rods onto the posterior surface of the sacrum with abundant bone graft extending from the lumbar spine onto the sacrum and fully covering the ends of the rods. Doubled sublaminar wires extend to the L_5_–S_1_ interspace but not beyond. We report here on the sacral rod/bone graft onlay stabilization technique.

## Materials and methods

### Surgical technique and post-operative management

Two operative procedures were used. Group 1 (14 patients): Harrington rod-Luque L rod extending onto sacrum with bone graft. A single Harrington rod with 2 hooks, the upper one in the thoracic region and the lower under L_5_, was supplemented with a second rod (Luque L rod) with its distal transverse component positioned distal to the lumbar hook resting on the sacrum, with both rods stabilized by sublaminar wires. Abundant bone graft, using both autograft bone from the spinous processes and allograft cortico-cancellous bone, was continued beyond L_5_ onto the sacrum completely covering the distal ends of the H-rod and hook and L-rod. Group 2 (51 patients): two Luque L rods or two straight rods extending onto the sacrum with bone graft. Two 4.5- or 5.5-mm-diameter rods were used with stabilization provided by 2 doubled #16 gauge sublaminar wires at each level, one holding each rod. Cross-links were used along with contouring of the rods to the lordotic position and placement of abundant autograft and allograft bone from the lumbar region lateral to the two rods passing onto the sacrum and fully covering the distal ends of the rods. All 65 surgical cases were operated by the senior author. Each patient had an anterior–posterior (“clamshell”) orthosis made immediately post-surgery for use in the sitting position for 8–12 weeks to minimize post-operative discomfort and enhance sitting balance. Continuous bed rest was not part of the post-operative regimen and all patients resumed wheelchair seating in brace within 2–3 days of surgery. All patients were fully wheelchair-dependent at time of surgery. All patients had intraoperative spinal cord monitoring.

### Assessment of scoliosis and pelvic obliquity

Scoliosis and pelvic obliquity were assessed by sitting anteroposterior full spine radiographs immediately before surgery, within a few weeks post-surgery, and at subsequent clinic visits post-surgery. The Cobb method was used to measure scoliosis. Pelvic obliquity was measured by determining the angle between a line drawn from the superior surfaces of the right and left iliac crests and the horizontal line at the bottom of the sitting radiograph. For the study of scoliosis and pelvic obliquity, measurements in all patients pre-surgery and within a few weeks post-surgery were used, and, in those having radiographic follow-up for 2 years or longer, the final or most recent measurements were used. Mean values in each group and for the entire series were calculated. This enabled us to assess the extent of correction of scoliosis and pelvic obliquity immediately after surgery, maintenance or loss of correction at 2 years or longer after surgery, and the amount of correction in relation to the extent of pre-operative deformity.

### Assessment of lumbosacral fusion

Bone fusion from the lower lumbar region to the sacrum was assessed on the anteroposterior and lateral full spine radiographs. In many patients, additional supine lumbosacral/pelvic radiographs in anteroposterior, lateral, and oblique projections further clarified the fusion mass.

### Additional analysis of patients

Other assessments included: age at surgery, the underlying diagnosis, male/female distribution, clinical outcome, and complications.

## Results

Diagnoses in the patients are listed in Table 1. None of the DMD patients in this study were on oral steroid treatment. The mean age at surgery was 13.0 years (±1.5, range 10.9–16.7) in Group 1; 14.6 years (±2.2, range 11.1–25.2) in Group 2; and 14.3 years (±2.2, range 10.9–25.2) for the entire study. The male:female distribution was 59:6 owing to the preponderance of patients with DMD which occurs only in males. Follow-up in patients assessed 2 years post-surgery or longer with radiographs was 7.6 years (±4.5, range 2–13.8) in Group 1; 6.1 years (±4.4, range 2–25.3) in Group 2; and 6.4 years (±4.4, range 2–25.3) for both groups together.

**Table 1:** Diagnoses of patients undergoing posterior spinal fusion to sacrum using sacral rod/bone graft onlay method

	Group 1	Group 2	Total
Duchenne muscular dystrophy	14	39	53
Other muscular dystrophies			
Limb girdle		2	2
Infantile FSH MD		1	1
Myopathy		3	3
Spinal muscular atrophy		4	4
Hypotonic cerebral maldevelopment			
Lissencephaly		1	1
Friedreich ataxia		1	1
Total	14	51	65

**Fig. 1:**
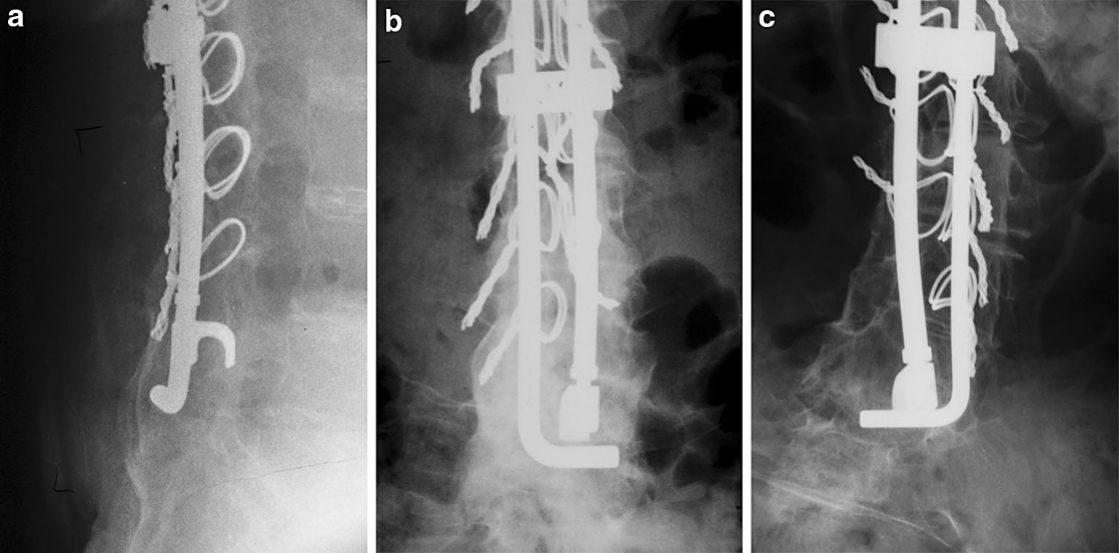
Radiographs illustrating lumbosacral fusions in Group 1 patients having Harrington rod–Luque L rod stabilization with sublaminar wires holding both rods. **a** Lateral radiograph in patient with DMD shows abundant bone continuous from lumbar region to sacrum completely encasing the distal ends of the two rods. **b** Anteroposterior radiograph in same patient as **a** shows continuous bone from lumbar vertebrae to sacrum. **c** Anteroposterior radiograph shows bone continuity from lumbar vertebrae to sacrum on both sides of vertebrae

**Fig. 2:**
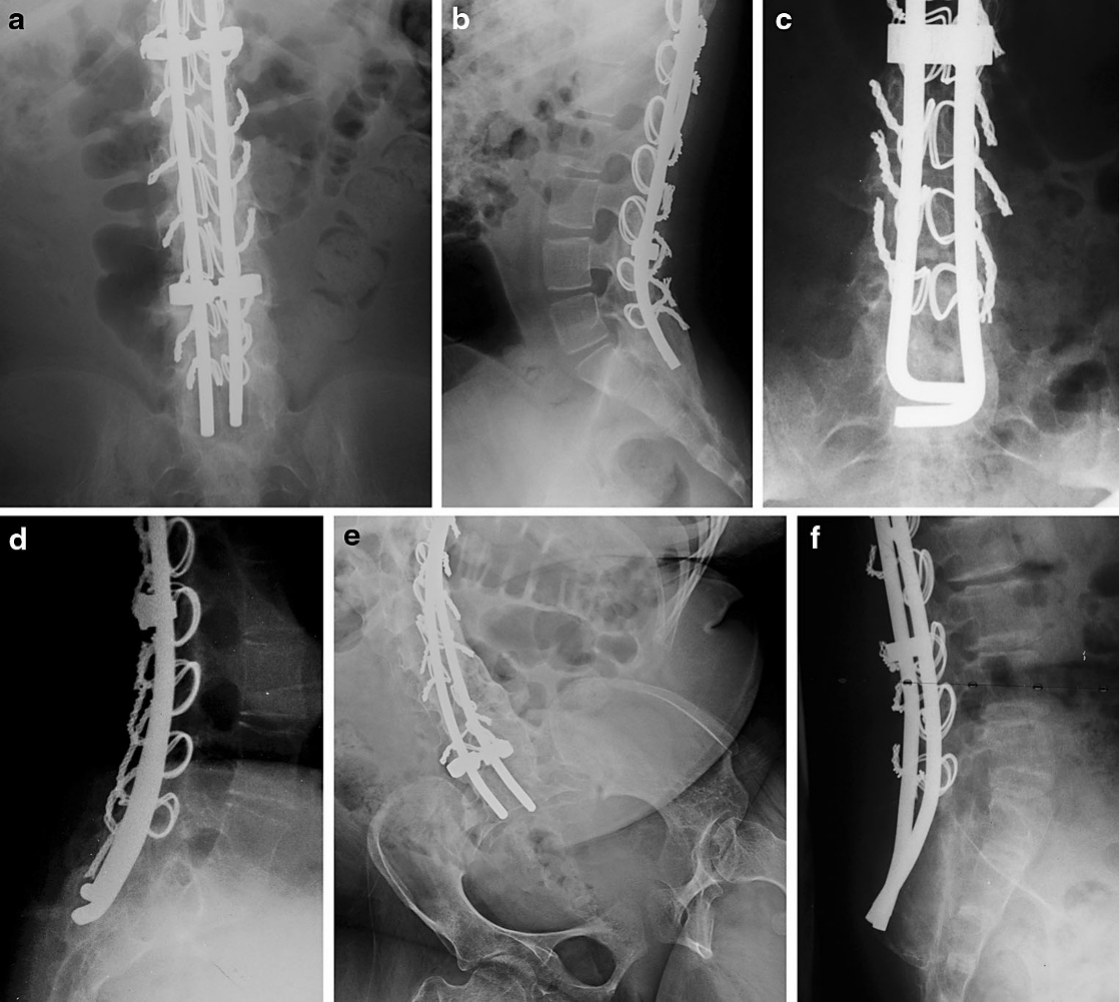
Radiographs illustrating lumbosacral fusions in Group 2 patients having double rod stabilization with sublaminar wire fixation. **a** Anteroposterior radiograph in patient with DMD illustrating lumbosacral bone continuity visible on outer sides of both rods. **b** Lateral radiograph in the same patient shown in **a** shows abundant bone from posterior lumbar region to the sacrum encasing the two rods. **c** Anteroposterior radiograph in a patient with DMD shows bony continuity from lumbar region to sacrum best seen lateral to each of the two rods. **d** Lateral radiograph from same patient shown in **c** shows lumbosacral bone continuity. Lordosis has been built into the rod positions. **e** Anteroposterior radiograph in patient with spinal muscular atrophy type 2 shows bony continuity from lumbar region to sacrum even though pelvic obliquity remained extensive. Note the denser bone continuity along the concavity of the lumbosacral curve. **f** Lateral radiograph shows extensive lumbosacral fusion even though the rod had not been contoured to lie against the posterior surface of the sacrum. Note the abundant bone distally between the two rods and the posterior surface of the sacrum

**Fig. 3:**
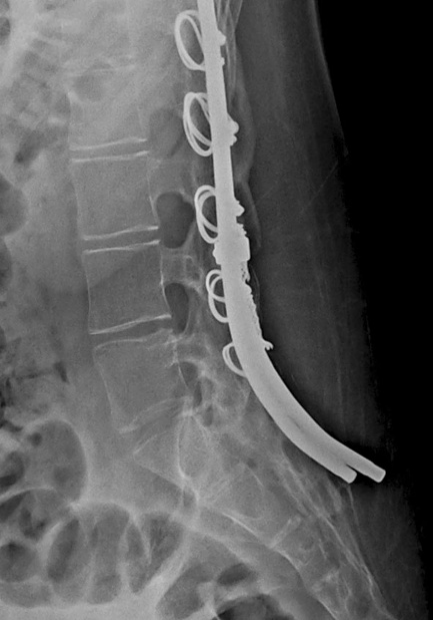
Lateral radiograph in patient with DMD shows rods that were too long and not firmly positioned against the posterior surface of the sacrum. Relief occurred after shortening of the rod ends. Note the solid continuous posterior bone fusion from the lumbar vertebrae across the lumbosacral joint onto the sacrum

Radiographs at final or most recent follow-up showed intact rods with maintenance of original position and most showed abundant bone fusion passing from the lower lumbar region onto the sacrum in antero-posterior, lateral, and oblique projections. The lumbosacral bone was often seen to completely cover the ends of the rods. In both groups, X-rays show extensive bone continuity from the lower lumbar region onto the sacrum on multiple projections (Figs. 1, 2, 3). In one instance, where CT scanning was done in an effort to determine the cause of pain, abundant continuous cortical bone was noted from the lumbar region to the sacrum on sagittal plane images. At operative intervention, continuous bone fusion encasing the rod was confirmed. Lumbosacral fusion was noted to occur regardless of the degree of lumbar scoliosis or pelvic deformity persisting after completion of the intraoperative surgical stabilization. Radiolucent regions around the ends of the rods in the sacral region (“windshield wiper” effect) were very infrequent, and, on the lateral radiograph, were covered and contained by cortical bone. There was no radiographic evidence of fracturing of rods or pseudarthrosis at the lumbosacral region in the entire series. Solid lumbosacral union occurred even in the more extensive scoliotic deformities with marked pelvic obliquity which were relatively minimally corrected. All patients remained comfortable in the sitting position several years post-surgery, once the prominent longer rods were revised (see below). There were no instances of motor or sensory nerve problems associated with the sublaminar wires or deformity correction.

The mean scoliosis deformity at the time of surgery was 54.7° (±31.1, range 0°–120°) with post-operative correction to a mean of 21.8° (±21.7, range 0°–91°) (correction of 60.1 %) and long-term maintenance of correction at a mean of 24.0° (±22.9, range 0°–94°) (correction of 56.1 %). The loss of scoliosis correction was only a mean of 2.2°. The mean pre-operative pelvic obliquity was 17.3° (±11.3, range 0°–51°) with post-operative correction to a mean of 8.9° (±7.8, range 0°–35°) (correction of 48.6 %) and long-term maintenance of correction at a mean of 10.1° (±8.1, range 0°–27°) (correction of 41.6 %). The loss of pelvic obliquity correction was only a mean of 1.2° (Table 2). Table 3 outlines the mean amount and percentage correction of deformity in relation to the extent of pre-operative deformity. For scoliosis in the range of 0°–40°, an 87.5 % correction was achieved; from 41° to 60°, a 67.0 % correction; and from 61° or greater, a 49.0 % correction. Pelvic obliquity from 0° to 10° pre-operatively had a 41 % correction; from 11° to 20°, a 55 % correction; and from 21° or greater, a 44 % correction.

**Table 2:** Scoliosis and pelvic obliquity measurements at pre-operative, post-operative, and long-term follow-up time periods

	Pre-operative	Post-operative	Long-term follow-up
**Scoliosis**			
Group 1			
Mean	42 (±33.44)	13.5 (±17.79)	14.8 (±18.85)
Range	1–100	0–45	0–45
* n*	14	13	8
Group 2			
Mean	58.2 (±29.79)	24.1 (±22.24)	26.1 (±23.37)
Range	0–120	0–91	0–94
* n*	51	50	39
Both			
Mean	54.7 (±31.07)	21.8 (±21.68)	24.0 (±22.86)
Range	0–120	0–91	0–94
% correction		60.1	56.1
* n*	65	63	47
**Pelvic obliquity**			
Group 1			
Mean	15.4 (±10.7)	6.2 (±5.8)	6.8 (±8.4)
Range	0–32	0–15	0–25
* n*	12	11	7
Group 2			
Mean	7.8 (±11.5)	9.2 (±8.2)	10.8 (±8.0)
Range	0–51	0–35	0–27
* n*	45	43	36
Both			
Mean	17.3 (±11.3)	8.9 (±7.8)	10.1 (±8.1)
Range	0–51	0–35	0–27
% correction		48.6	41.6
* n*	57	54	43

**Table 3:** Correction of scoliosis and pelvic obliquity based on extent of pre-operative deformities

	Average pre-operative	Average post-operative	% correction
**Scoliosis**			
Minimal deformity (0°–40°)	23.21 (±10.31)	2.92 (±4.67)	87
Moderate deformity (41°–60°)	52.84 (±5.56)	18.37 (±11.15)	67
Severe deformity (61°+)	90.68 (±17.53)	45.45 (±17.25)	49
**Pelvic obliquity**			
Minimal deformity (0°–10°)	4.31 (±3.07)	2.54 (±2.22)	41
Moderate deformity (11°–20°)	14.86 (±3.31)	6.71 (±4.22)	55
Severe deformity (21°+)	29.84 (±8.20)	16.63 (±9.67)	44

### Complications

In the entire group of 65 patients, 5 developed pain at the distal ends of the rods requiring surgical revision. Each of the symptomatic patients was in Group 2. In those patients requiring revision to shorten the rod at the sacral region, the excessive length of the rods at time of insertion and their lack of close coaptation to the posterior sacral bone explain the subsequent problems (Fig. 3). Four of the five surgical revisions for pain were due to prominence of the distal ends of the rods at the sacral level. Each of these four (all with DMD) was successfully managed with local exploration, shortening of the rods by a length of 2–3 cm, and, if still necessary, bending of the rods to lie firmly against the sacrum. One of these had associated skin breakdown and sepsis at the distal site which healed after rod shortening. The fifth patient (limb girdle muscular dystrophy) benefited from surgical shortening of the distal end of one rod on the painful side, even though it was not prominent subcutaneously and relief was due to associated release of foraminal nerve pressure at L_5_. Prior to the revision, the patient had three lumbo-sacral region injections by the Pain Service, each with only a few months relief. We noted that four of the five patients had a significant weight loss between the time of original surgery and the time of rod prominence and local discomfort. In these patients, the mean weight loss was 13.8 kg (range 10–17 kg) and mean time following original surgery was 4.4 years (range 1.5–7.0 years). The weight loss and time after initial surgery in the four patients were: 17 kg (71–54 kg), 1.5 years; 13 kg (62–49 kg), 3.3 years; 10 kg (54–44 kg), 4.1 years; and 15 kg (61–46 kg), 7.0 years. One patient had gained 13 kg (85–98 kg) at time of revision at 0.8 years. In each of the five cases, the rods did not migrate from the bone but were in retrospect relatively too long and not positioned closely against the sacrum at time of initial surgery.

## Discussion

Many patients with DMD and other non-ambulatory hypotonic neuromuscular disorders have had posterior spinal fusion for scoliosis over the past few decades. In an early subset of 26 procedures in our unit for DMD, 17 had a Harrington rod–Luque rod construct with both rods stabilized by 2 doubled #16 gauge sublaminar wires at each level and 1 or 2 cross-links [26]. In most of these cases, the rods were placed only to the lower lumbar region, but in 4, the instrumentation and bone graft extended to the sacrum and solid lumbosacral fusion was noted to occur. We continued to use this latter approach with good results. Patients were stabilized to the sacrum with the Luque rod transverse bar placed onto the sacrum distal to the L_5_–S_1_ joint, with abundant autograft and allograft bone also spanning the L_5_–S_1_ space onto the sacrum (Group 1). Longer-term studies continued to show excellent lumbosacral fusion and a good comfort level. The procedure extending the fusion to the sacrum was then used in all non-ambulatory hypotonic neuromuscular scoliosis procedures when two straight rods with sublaminar wires and cross-links replaced the Harrington–Luque construct (Group 2). In a few instances where we attempted placement of sacral and iliac screws, they did not lead to meaningful stabilization owing to the softness of the osteopenic bones. On occasion, in patients we saw from other centers, the longer pelvic screws were not fully contained within bone. The osteopenia and often small and misshapen pelvic structure in these severely involved neuromuscular patients led us to continue with the sacral rod/bone graft-onlay approach.

The stabilization procedure was used regardless of the extent of scoliosis deformity or pelvic obliquity. However, the scoliosis and pelvic obliquity were always improved in the operated patients as a result of (1) the intra-operative prone position, (2) specific attention to straightening the spine and pelvis with positioning on the scoliosis frame before starting surgery, (3) the primary thoracolumbar scoliosis correction that occurred with rod, hook, and sublaminar wire instrumentation, and (4) the passive pelvic correction that accompanied the lumbar straightening.

Radiographic continuity of bone across the lumbosacral joint in anteroposterior, lateral, and oblique projections and the absence of discomfort were considered to indicate a good result following extension of the instrumentation and fusion to the sacrum. There was no change in the position of the rods. The discomfort that developed in a few was due to the prominence of the distal ends of the rods worsened by excessive weight loss (and thus less soft tissue coverage). No rods fractured or pulled away from the bone post-surgery in the entire series. The lumbo-sacral bone fusion was clearly demonstrated by radiographs (Figs. 1, 2, 3). In the one patient who had unilateral exploration of the distal rod at the site of persistent pain 3.3 years post-surgery, with no radiographic or clinical evidence of subcutaneous prominence, there was full fusion of the operative (bone) mass down to and including the sacrum, and the distal ends of both rods were encased in mature thick cortical bone. CT scans just prior to exploration supported this observation. The “windshield wiper” effect was seen radiographically very infrequently in our patients. It has been noted more extensively in other methods where the rods are placed for longer distances within sacral and iliac bone. The marked immobility of our patient population contributes to the absence of this finding.

The measurements of scoliosis and pelvic obliquity pre-surgery and post-surgery show good correction of both parameters with this technique, and the correction is well maintained with the longer-term follow-up (Table 2). These measurements along with the radiographic appearances and clinical findings (of comfortable seating) appear to indicate that the sacral rod/bone graft-only technique induces stable lumbosacral fixation in this specific subset of non-ambulatory very weak hypotonic neuromuscular patients. The “windshield wiper” radiologic finding of bone lysis around the distal ends of the rods was very infrequent in this series, also indicating good stability and fusion.

Virtually all studies of neuromuscular scoliosis surgery in early adolescence show a favorable post-operative correction with a slight to moderate worsening with time (although not to a clinically significant extent). Gaine et al. [35] showed this clearly in a large series of cases with differing techniques and also with fusion to L_5_ or to the sacrum. Our findings are similar to other series with only a minimal 2.2° loss of correction for scoliosis. A similar pattern is seen regarding pelvic obliquity. The loss of correction of only 1.2° in this series is also a very favorable finding.

The tendency in spinal deformity surgery with stabilization to L_5_ or to the pelvis in neuromuscular patients (most of whom are non-ambulatory) is to get very good initial correction of both scoliosis and pelvic obliquity with a slight loss of correction over the next several years. Examples reporting mean values pre-operatively, post-operatively, and at latest follow-up include: (Cotrel–Dubousset to pelvis, 18 patients) scoliosis 70° to 38° to 41°; pelvic obliquity 19° pre-operatively in 13, 9 improved 22° to 11°, and 4 worsened 13° to 16° [10]; (Harrington–Luque, most Luque to pelvis with modified Moe fusion, 101) scoliosis 84° to 40° with mean loss of correction of 7°; pelvic obliquity 21° to 11° with mean loss of correction of 3° [29]; (Luque rod/Galveston, 31) scoliosis 48° to 16.7° to 22°; pelvic obliquity 19.8° to 7.2° to 11.6° [30]; [Luque single unit rod, pedicle screws, 74 (25 to sacrum or pelvis)] scoliosis 53.5° to 27.3° to 39°; pelvic obliquity 20° to 10.8° to 16° [35]; (pedicle screws and iliac screws, 20) scoliosis 44° to 10°; pelvic obliquity 14° to 3° [14]; (Jackson intrasacral fixation, hybrid above, 56) scoliosis 58.5° to 22.3° to 23.5°, pelvic obliquity-improved [15]; (sublaminar wires group A, sublaminar wires and pedicle screws group B, pedicle screws group C, 43) scoliosis changes group A: 50° to 15.7° to 21.6°, group B: 17.8° to 3.6° to 6.7°, and group C: 25.8° to 5.5° to 8.9° [33]; (pedicle screw instrumentation, 27; iliac screws to pelvis, 18) scoliosis (entire 27) 79.8° to 30.2° to 31.9°; pelvic obliquity for cases extended to pelvis 22.2° to 11.2° to 13.4° [21]; (Luque–Galveston, 93) scoliosis 72° to 33° to 36° [20]; (new pelvic rod system, 18) scoliosis 82.3° to 30.9° to 33.4°; pelvic obliquity 19.3° to 5° to 5° [6].

Many recommend fusion to the pelvis in all non-ambulatory patients having spinal fusion [6, 15, 17, 18, 29], while those fusing only to L_5_ recommend doing so in those with early or milder deformities such as scoliosis <40°, pelvic obliquity <10° (or 15°) and the apex of the curve at L_1_ (or L_2_) and above [21, 25, 27, 30, 34]. Alman and Kim [27] fused 38 DMD patients to L_5_ but noted subsequent increase of pelvic obliquity in all of at least 10°, while none of 10 fused to the pelvis showed any increase. Takaso et al. [34] fused 28 patients with DMD to L_5_ only in those with the scoliosis apex at L_2_ or higher and preferably with a minimal L_5_ tilt <15°. They decreased the pre-operative mean 74° curves to 14° post-operatively and 17° at latest follow-up, with pelvic obliquity at 17° pre-operatively and 6° post-operatively and at latest follow-up. Gaine et al. [35] felt that fusion to S_1_ (sacrum) did not provide any benefit over fusion to L_5_ with regard to correction and maintenance of both parameters.

We noted lesser corrections of scoliosis as the pre-operative deformity increased, as the greater curves had usually developed over longer periods of time and were more rigid. The smallest curves (0°–40°) had a correction of 87.5 % while the largest curves (61°+) had only a 49 % rate of correction. This was not true for pelvic obliquity even though it might have been expected, but perhaps the small angles involved were less easily documented. Decreased percent correction in larger curves >70° was also noted by Bentley et al. [29].

We stress that the sacral rod/bone graft onlay procedure is indicated and effective only in non-ambulatory neuromuscular patients where the stresses placed on the lumbosacral junction are minimal. We do not recommend this approach for ambulatory patients or for patients who self-transfer from bed to chair where rotational stresses can be considerable. It is particularly warranted for those with some or all of moderate to severe osteoporosis, relatively small stature, and considerable pelvic bone deformation.

Several technical features lead to a more effective outcome: (1) it is necessary to bend both rods at the lumbosacral region into a lordotic conformation; (2) the distal rods must be placed tightly against the sacral bone surface, however, with the most distal stabilization by the sublaminar wires at the L_5_–S_1_ level; (3) thoracic and lumbar cross-links are helpful to further limit movement of the two rods; cross-links should not be attached over the sacrum if possible; (4) the rods should extend onto the sacrum for a relatively short distance of 2–3 cm beyond the L_5_–S_1_ joint to minimize the likelihood of becoming prominent with time; (5) the sacrum must be extensively cleared in the subperiosteal plane including for a few centimeters distal and lateral to the ends of the rods and abundant autograft (spinous processes) and allograft (cortico-cancellous) bone graft should be used completely covering the two rods in the lower lumbar and sacral position; (6) since weight loss and further muscle atrophy are common in many progressive neuromuscular disorders in late adolescence and early adulthood, soft tissue coverage of the instrumentation at time of surgery is extremely important since it may diminish significantly with time; (7) attention is paid to straightening the pelvis by pre-operative positioning on the scoliosis frame and application of the posterior instrumentation for optimal scoliosis and particularly lumbar scoliosis correction; and (8) use of an anterior–posterior brace post-surgery in the sitting position for several weeks is part of our management program. One clear limitation of the procedure is its inability to primarily actively tilt or reposition any persisting pelvic obliquity after the above-mentioned approaches. Fusion occurred in all cases, however, regardless of persisting lumbar scoliosis or pelvic obliquity and these parameters did not subsequently worsen.

The spinal rod/bone graft onlay technique has proven to be valuable in this small subset of non-ambulatory patients with hypotonic neuromuscular diseases having posterior spinal fusion for scoliosis in adolescence. The use of steroids as a treatment for DMD has dramatically diminished the need for spinal fusion from 90 % pre-steroids to as low as 20 % with long-term steroid use [36]. However, when surgery is needed in patients on long-term steroids, osteoporosis is an even greater problem. A considerable number of patients with DMD are not treated with steroids long term owing to parental or medical provider decision or medical contra-indications such as excess weight gain, development of severe osteoporosis with fractures and bone pain, glaucoma, and worsening behavioral activity. Increasing numbers of patients with DMD and types 1 and 2 spinal muscular atrophy are surviving with earlier use of longevity enhancing measures such as g-tubes for nutrition, part-time respiratory support with BiPAP, and full-time support with tracheostomy and mechanical ventilators. Large neuromuscular clinics also see increasing numbers of wheelchair-dependent children and adolescents most of whom develop a progressive scoliosis with other disorders, such as the myopathies, non-dystrophin-related muscular dystrophies, and Friedreich ataxia. While all our cases had sublaminar wires, this technique can also be used for hook- or pedicle screw-based systems; hooks or screws would extend only to the L_5_ level while the rods and abundant bone graft extend distally onto the sacrum.

## References

[1] Moshirfar A, Rand F, Sponseller P, Parazin S, Khanna A, Kebaish K, Stinson J, Riley L (2005). Pelvic fixation in spine surgery: historical overview, indications, biomechanical relevance, and current techniques. J Bone Joint Surg.

[2] Hibbs RA, Risser JC, Ferguson AB (1931). Scoliosis treatment by the fusion operation. An end result study of three hundred and sixty cases. J Bone Joint Surg.

[3] Smith A, Butte FL, Ferguson AB (1938). Treatment of scoliosis by the Wedging Jacket and spine fusion. A review of 265 cases. J Bone Joint Surg.

[4] Thompson WAL, Ralston EL (1949). Pseudarthrosis following spine fusion. J Bone Joint Surg (Am).

[5] Kebaish KM (2010). Sacropelvic fixation: techniques and complications. Spine.

[6] Chechik O, Fishkin M, Weintroub S, Ovadia D (2011). A new pelvic rod system for the surgical correction and fixation of pelvic obliquity in pediatric neuromuscular scoliosis. J Child Orthop.

[7] Allen B, Ferguson RL (1984). The Galveston technique of pelvic fixation with L-rod instrumentation of the spine. Spine.

[8] Kornblatt M, Casey M, Jacobs R (1986). Internal fixation in lumbosacral spine fusion. A biomechanical and clinical study. Clin Orthop Rel Res.

[9] McCarthy RE, Dunn H, McCullough FL (1989). Luque fixation to the sacral ala using the Dunn–McCarthy modification. Spine.

[10] Neustadt J, Shufflebarger H, Cammisa FP (1992). Spinal fusions to the pelvis augmented by Cotrel–Dubousset instrumentation for neuromuscular scoliosis. J Pediatr Orthop.

[11] Miladi L, Ghanem I, Draoui M, Zeller R, Dubousset J (1997). Iliosacral screw fixation for pelvic obliquity in neuromuscular scoliosis: a long-term follow-up study. Spine.

[12] Tsuchiya K, Bridwell K, Kuklo T, Lenke L, Baldus C (2006). Minimum 5-year analysis of L_5_–S_1_ fusion using sacropelvic fixation (bilateral S_1_ and iliac screws) for spinal deformity. Spine.

[13] Peelle M, Lenke LG, Bridwell K, Sides B (2006). Comparison of pelvic fixation techniques in neuromuscular spinal deformity correction: Galveston rod versus iliac and lumbosacral screws. Spine.

[14] Hahn F, Hauser D, Espinosa N, Blumenthal S, Min K (2008). Scoliosis correction with pedicle screws in Duchenne muscular dystrophy. Eur Spine J.

[15] Ilharreborde B, Hoffmann E, Tavakoli S, Queinnec S, Fitoussi F, Presedo A, Pennecot GF, Mazda K (2009). Intrasacral rod fixation for pediatric long spinal fusion: results of a prospective study with a minimum of 5-year follow-up. J Pediatr Orthop.

[16] Chang TL, Sponseller PD, Kebaish KM, Fishman EK (2009). Low profile pelvic fixation: anatomic parameters for sacral alar-iliac fixation versus traditional iliac fixation. Spine.

[17] Zahi R, Vialle R, Abelin K, Mary P, Khouri N, Damsin JP (2010). Spinopelvic fixation with iliosacral screws in neuromuscular spinal deformities: results in a prospective cohort of 62 patients. Childs Nerv Sys.

[18] Zahi R, Thévenin-Lemoine C, Rogier A, Constantinou B, Mary P, Vialle R (2011). The “T-construct” for spinopelvic fixation in neuromuscular spinal deformities, preliminary results of a prospective series of 15 patients. Childs Nerv Syst.

[19] Ramirez N, Richards S, Warren P, Williams G (1997). Complications after posterior spinal fusion in Duchenne’s muscular dystrophy. J Pediatr Orthop.

[20] Lonstein JE, Koop SE, Novachek TF, Perra JH (2012). Results and complications after spinal fusion for neuromuscular scoliosis in cerebral palsy and static encephalopathy using Luque Galveston instrumentation: experience in 93 patients. Spine.

[21] Modi HN, Suh SW, Hong JY, Cho JW, Park JH, Yang JH (2010). Treatment and complications in flaccid neuromuscular scoliosis (Duchenne muscular dystrophy and spinal muscular atrophy) with posterior-only pedicle screw instrumentation. Eur Spine J.

[22] Linam WM, Margolis PA, Staat MA, Britto MT, Hornung R, Cassedy A, Connelly BL (2009). Risk factors associated with surgical site infection after pediatric posterior spinal fusion procedure. Infect Control Hosp Epidemiol.

[23] McCord DH, Cunningham BW, Shono Y, Myers JJ, McAfee PC (1992). Biomechanical analysis of lumbosacral fixation. Spine.

[24] Early S, Mahar A, Oka R, Newton P (2005). Biomechanical comparison of lumbosacral fixation using Luque–Galveston and Coloardo II sacropelvic fixation: advantage of using locked proximal fixation. Spine.

[25] Mubarak S, Morin W, Leach J (1993). Spinal fusion in Duchenne muscular dystrophy—fixation and fusion to the sacropelvis. J Pediatr Orthop.

[26] Shapiro F, Sethna N, Colan S, Wohl ME, Specht L (1992). Spinal fusion in Duchenne muscular dystrophy: a multidisciplinary approach. Muscle Nerve.

[27] Alman BA, Kim HKW (1999). Pelvic obliquity after fusion of the spine in Duchenne muscular dystrophy. J Bone Joint Surg (Br).

[28] Westerlund L, Sanjitpal S, Jarosz T, Abel M, Blanco J (2001). Posterior-only unit rod instrumentation and fusion for neuromuscular scoliosis. Spine.

[29] Bentley G, Haddad F, Bull TM, Seingry D (2001). The treatment of scoliosis in muscular dystrophy using modified Luque and Harrington-Luque instrumentation. J Bone Joint Surg (Br).

[30] Sengupta DK, Mehdian SH, McConnell JR, Eisenstein SM, Webb JK (2002). Pelvic or lumbar fixation for the surgical management of scoliosis in Duchenne muscular dystrophy. Spine.

[31] Miller F, Moseley C, Koreska J (2008). Spinal fusion in Duchenne muscular dystrophy. Dev Med Child Neurol.

[32] McCall RE, Hayes B (2005). Long-term outcome in neuromuscular scoliosis fused only to lumbar 5. Spine.

[33] Arun R, Srinivas S, Mehdian SMH (2010). Scoliosis in Duchenne’s muscular dystrophy: a changing trend in surgical management. Eur Spine J.

[34] Takaso M, Nakazawa T, Imura T, Ueno M, Saito W, Shintani R, Takahashi K, Yamazaki M, Ohtori S, Okamoto M, Masaki T, Okamoto H, Okutomi T, Ishii K, Ueda Y (2010). Can the caudal extent of fusion in the surgical treatment of scoliosis in Duchenne muscular dystrophy be stopped at lumbar 5?. Eur Spine J.

[35] Gaine WJ, Lim J, Stephenson W, Galasko CSB (2004). Progression of scoliosis after spinal fusion in Duchenne’s muscular dystrophy. JBJS (Br).

[36] Lebel DE, Corston JA, McAdam LC, Biggar WD, Alman A (2013). Glucocorticoid treatment for the prevention of scoliosis in children with Duchenne muscular dystrophy: long-term follow-up. J Bone Joint Surg Am.

